# High Serum Alkaline Phosphatase, Hypercalcaemia, Race, and Mortality in South African Maintenance Haemodialysis Patients

**DOI:** 10.1155/2017/2795432

**Published:** 2017-01-12

**Authors:** Bala Waziri, Raquel Duarte, Saraladevi Naicker

**Affiliations:** Department of Internal Medicine, Faculty of Health Sciences, University of the Witwatersrand, Johannesburg, South Africa

## Abstract

*Objective.* To determine the association between serum total alkaline phosphatase (TAP) and mortality in African maintenance haemodialysis patients (MHD).* Patients and Methods.* The study enrolled a total of 213 patients on MHD from two dialysis centers in Johannesburg between January 2009 and March 2016. Patients were categorized into a low TAP group (≤112 U/L) versus a high TAP group (>112 U/L) based on a median TAP of 112 U/L.* Results.* During the follow-up period of 7 years, there were 55 (25.8%) deaths. After adjusting for cofounders such as age, other markers of bone disorder, and comorbidity (diabetes mellitus), patients in the high TAP group had significantly higher risk of death compared to patients in the low TAP group (hazard ratio, 2.50; 95% CI 1.24–5.01,* P *= 0.01). Similarly, serum calcium >2.75 mmol/L was associated with increased risk of death compared to patients within levels of 2.10–2.37 mmol/L (HR 6.34, 95% CI 1.40–28.76;* P *= 0.02). The HR for death in white patients compared to black patients was 6.88; 95% CI 1.82–25.88;* P* = 0.004.* Conclusion.* High levels of serum alkaline phosphatase, hypercalcaemia, and white race are associated with increased risk of death in MHD patients.

## 1. Introduction

Prior to the availability of commercial intact parathyroid hormone (PTH) assays, serum total alkaline phosphatase (TAP) measurements were used as one of the surrogate markers of high bone turnover that was utilized in the management of chronic kidney disease mineral and bone disorder (CKD-MBD) [1]. Subsequently, in 2003 the Kidney Disease Outcome Quality Initiative (KDOQI) guidelines on CKD-MBD made no recommendations regarding the use of alkaline phosphatase and this has made it a less preferred marker to PTH. However, in 2009 the Kidney Disease Improving Global Outcomes (KDIGO) guidelines recommended measurement of TAP every 12 months in CKD 4-5D [2] and more recently evidence continued to emerge on the importance of higher levels of alkaline phosphatase in the pathogenesis of vascular calcification via hydrolysis of pyrophosphate which is a potent inhibitor of vascular calcification [3–5]. This was further supported by a study that showed elevated levels of alkaline phosphatase, independent of PTH, calcium, or phosphorus as predictor of coronary artery calcification in haemodialysis patients [6]. Interestingly, in a recent secondary analysis of the handling erythropoietin resistance with oxypentifylline (HERO) trial, high levels of alkaline phosphatase were also associated with erythropoietin stimulating agent hyporesponsiveness [7]. These findings may likely explain the unclear pathophysiologic link between high serum alkaline phosphatase and mortality in haemodialysis patients [6].

Although the role of racial disparities in adverse clinical outcomes remains controversial and inconclusive, some studies have demonstrated survival benefits attributable to race in patients undergoing MHD [8, 9]. In addition, the impact of these biochemical abnormalities have been shown to differ across race and thus the need for race specific target values for these markers of mineral bone disorder [10, 11].

Therefore, the aim of this study was to determine if there is a link between high serum alkaline phosphatase and mortality in African MHD patients.

## 2. Patients and Methods

This study was a retrospective review of patients undergoing MHD from two dialysis centers in Johannesburg between January 2009 and March 2016. A total of 213 patients aged ≥ 18 years with available baseline line variables of interest were included. Exclusion criteria included patients with missing important data for analysis, being on dialysis for less than three months, having active or chronic liver disease, and having malignancies. In addition, we excluded Indian and mixed races to allow for a proper comparison between black and white patients. Retrieved data included patients' demographic characteristics, blood pressure measurements, duration on haemodialysis, comorbid disease, and medication history related to CKD-MBD. Determination of race was based on self-report by the participants.

Patients were categorized into the low TAP group (≤112 U/L) versus the high TAP group (>112 U/L) based on median TAP level of 112 U/L. Secondary analysis involved exploring the relationship between race, other markers of mineral bone disorder, and primary outcome. In line with a previous study [12] total calcium levels were categorized into four categories with the KDOQI target range as the reference category. Based on the KDIGO CKD-MBD guidelines, PTH was divided into three categories.

The primary outcome of this study was death and events other than death were censored and this included kidney transplantation, loss to follow-up, or still undergoing haemodialysis at the end of the study.

### 2.1. Laboratory Measurements

Patients' baseline biochemical parameters (within the first three months of initiating dialysis) were assessed. Most of the biochemical markers were measured monthly except for quarterly PTH. Plasma intact PTH was measured by an electrochemiluminescence immunoassay (ECLIA) run on a Cobas 6000 autoanalyzer (Roche Diagnostics, Mannheim, Germany; reference range 10–65 pg/mL). Serum 25-OH vitamin D was measured by a chemiluminescent microparticle immunoassay (CMIA) technique run on the ARCHITECT C8000 autoanalyzer (Abbott Laboratories, Abbott Park, IL, US). Reference ranges are as follows: <10 ng/mL as severe deficiency, 10–29 ng/mL as moderate deficiency, 30–100 ng/mL as sufficiency, and >100 ng/mL as toxic.

Serum calcium, phosphate, and alkaline phosphatase were measured using the ARCHITECT C8000 autoanalyzer (Abbot Laboratories, Abbott Park, IL, US). The corrected calcium was determined using the formula: corrected calcium (mmol/L) = calcium measured (mmol/L) + 0.02 [40-albumin (g/L)]. Total alkaline phosphatase reference range is 53–128 U/L.

Plasma albumin was measured by colorimetric (bromocresol green) method on a Cobas 6000 autoanalyzer (Roche Diagnostics, Mannheim, Germany; reference range 35–52 g/L).

Other biochemical parameters were determined using routine laboratory techniques.

Blood samples were generally collected predialysis at midweek with the exception of the postdialysis serum urea for kinetic modeling.

Calculation of normalized protein catabolic rate was based on the formula [13], nPCR = (0.136 × *F*) + 0.251, where *F* = Kt/V × ([predialysis BUN + postdialysis BUN] ÷ 2).

### 2.2. Statistical Analyses

Pearson's or Fisher's exact test was utilized for proportion comparisons. Continuous variables are presented as means ± standard deviations or median and interquartile range (IQR) as appropriate. Associations between serum alkaline phosphatase and other biochemical parameters were assessed by multiple linear regression analyses. The Cox proportional model was used to determine the crude and adjusted hazard ratios of death for different categories of serum alkaline phosphatase, calcium, PTH, phosphate, 25-OH vitamin D, and white versus black patients. Patients' demographic and baseline characteristics were compared between the low and high total alkaline phosphatase groups as well as white versus black patients, using an independent *t*-test and Mann–Whitney *U* test for normally distributed and nonnormally distributed variables, respectively. One-way ANOVA and Kruskal-Wallis tests were used to compare normally and nonnormally distributed continuous variables across categories of serum calcium.

A *P* value of less than 0.05 was considered statistically significant at the 95% confidence interval. All analyses were performed using STATA version 12 (STATA Corp., TX, USA).

## 3. Results

The study included two hundred and thirteen patients (137 men, 76 women) undergoing MHD. The mean (±SD) of age, median dialysis vintage, and mean Kt/V were 54.5 ± 15.6 years, 24 months (IQR, 12–48), and 1.44 ± 0.3, respectively. The majority of the patients were on three times weekly, 4 hr sessions of haemodialysis. Most of the patients were dialyzed with a dialysate calcium concentration of 1.50 mmol/L, which is usually modified based on serum levels of calcium. The blood and dialysis flow rates are generally 300–400 mls/min and 500 mls/min, respectively. However, these values varied according to patient's blood pressure and haemodynamic state. A native arteriovenous fistula was used in more than half of the study population (60.6%). Almost all patients (93.0%) were on ESAs.

Table 1 shows the comparisons of baseline clinical characteristics between patients in high TAP and low TAP groups. The low alkaline phosphatase group had significantly higher mean age than the high TAP group. Other parameters were comparable between the groups. For the management of CKD-MBD, 76.9% of the patients were on calcium carbonate and 64.3% on alfacalcidol with a similar distribution of drug usage across the groups. The study population included 120 (56.3%) black and 93 (43.7%) white patients. The mean age, hemoglobin concentration, albumin, and phosphate levels were significantly higher in white compared to black patients. 56 (26.3%) of the study population had diabetes and the proportion was higher in black patients (30.0% versus 21.5%, *P* = 0.02) (Table 2).

The characteristics of the patients across different categories of serum calcium levels are shown in Table 3. Patients in the highest category of calcium levels had significantly lower mean serum creatinine, and a few of them were on calcium carbonate and alfacalcidol. No significant differences were found in other parameters of the patients across the serum calcium categories. The overall mean dialysate calcium was 1.65 ± 0.24 mmol/L and patients with higher levels of calcium are more likely to be dialyzed with lower dialysate calcium concentration. To further explore our practice pattern regarding treatment of hypercalcaemia, available data revealed that only 5 patients in the highest category of calcium had undergone a parathyroidectomy while the majority of them were dialyzed with 1.25 mmol/L of dialysate calcium concentration and had their calcium carbonate and alfacalcidol discontinued.

During a follow-up period of 7 years there were 57 (26.8%) deaths. After adjusting for cofounders such as age, other markers of bone disorder (calcium, phosphate, and PTH), serum alanine transaminase, 25-OH vitamin D, and comorbidity (diabetes mellitus), patients in the high TAP group had a significantly higher risk of death compared to patients in the low TAP group (hazard ratio, 2.5; 95% CI 1.24–5.01, log rank *P* = 0.01).

**Figure 1:**
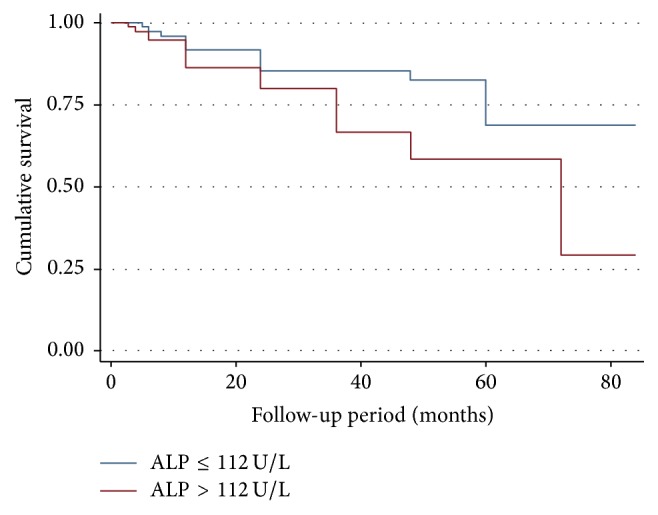
Kaplan Meier curve comparing patients in the high alkaline phosphatase to low alkaline phosphatase group (*P* = 0.01).

**Figure 2:**
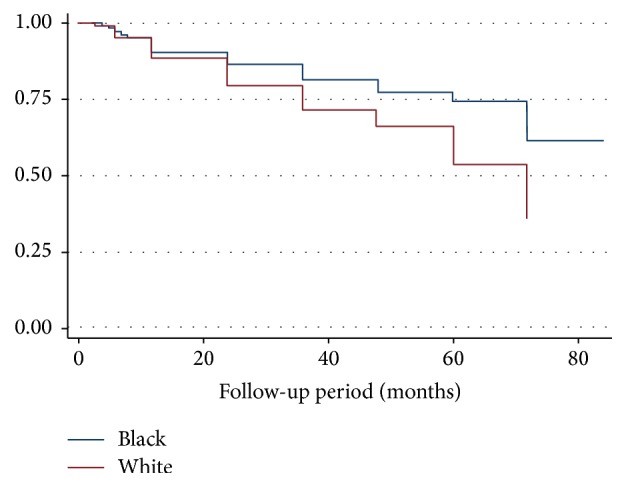
Kaplan Meier survival curve between black and white (*P* = 0.004).

**Figure 3:**
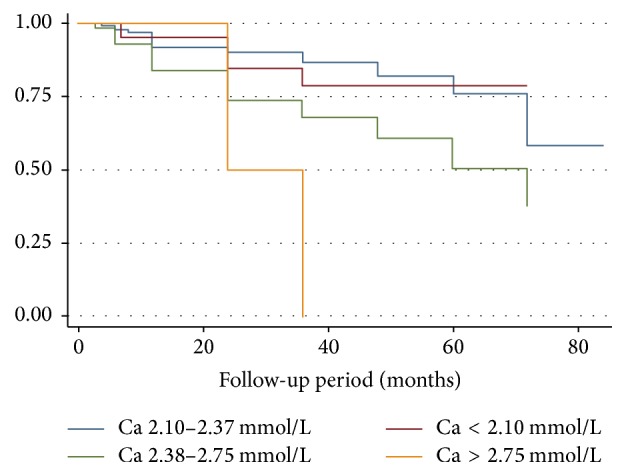
Kaplan Meier survival curves for different categories of calcium (*P* = 0.02). Calcium categories based on KDOQI reference range.

Patients in the highest category of corrected calcium (>2.75 mmol/L) had more than a sixfold increased risk of death compared to patients with normal calcium (HR 6.34, 95% CI 1.40–28.76; *P* = 0.02). Similarly, we found a significant association between race and mortality, in which white patients had an accentuated six fold increase in adjusted hazard ratio for death compared to black patients (HR 6.88, 95% CI 1.82–25.88; *P* = 0.004) (Table 4). Figures 1, 2, and 3 show Kaplan Meir Survival curves for TAP, race, and calcium levels, respectively.

Univariate linear regression analysis revealed a significant association between TAP and age (*r*^2^ = 0.04, *P* = 0.008), corrected calcium (*r*^2^ = 0.03, *P* = 0.04), and PTH (*r*^2^ = 0.04, *P* = 0.006). In multivariate regression analyses PTH and calcium remained significantly correlated with TAP, *P* = 0.006 and 0.04, respectively.

## 4. Discussion

Several studies from Europe, America, and Asia have consistently shown a linear relationship between high serum alkaline phosphate and mortality in the haemodialysis population [14–17], while results relating to other markers of mineral metabolism revealed nonlinear (U or J patterns) associations [12, 18, 19]. Such data relating to the impact of markers of CKD-MBD on mortality in African MHD patients are lacking. In this present study, higher levels of TAP, hypercalcaemia, and white race were associated with increased risk of death. These findings are consistent with other large studies where higher levels of TAP were independently associated with higher risk of mortality [14, 17].

Interestingly, this association was also reported in CKD patients as well as in the general population [20, 21]. The National Health and Nutrition Examination Survey (NHANES) data revealed an independent association between elevated levels of TAP and mortality in the general population [21]. This further supports the notion that TAP is more than a marker of high bone turnover and may be a reliable predictor of mortality.

The mechanisms for this association have been linked to enhanced vascular calcification by high levels of serum TAP through hydrolysis of pyrophosphate or activation of apatite crystal formation [22]. In addition to vascular calcification, elevated levels of TAP have been associated with high C reactive protein, insulin resistance, and 25-OH vitamin D deficiency [23–26]. In contrast to our study, we found no significant difference in the mean levels of 25-OH vitamin D between patients with high TAP and low TAP.

Despite the variations in the cut-off points for defining hypercalcaemia by various studies, hypercalcaemia has been consistently associated with increased risk for mortality in haemodialysis patients [12, 18, 27]. Consistent with our finding, a linear relation was observed between higher calcium categories and increased risk of death [12, 18]. In a large global representation of HD patients including the three phases of the dialysis outcomes and practice patterns study (DOPPSI, II and III) with 25,588 HD patients, calcium levels greater than 10.0 mg/dL (>2.5 mmol/L) were significantly associated with greater risk of all cause and cardiovascular mortality in both baseline and time dependent models [27]. The reasons for this consistent association could be linked to acceleration of arterial calcification by hypercalcaemia [28, 29]. Besides vascular calcification, high levels of calcium, but not high PTH, have been associated with poor mental health in MHD patients [30]. In contrast, studies relating to hypocalcaemia and risk of death have yielded contradictory reports. Lowrie and Lew [31] were the first to establish the association of increased mortality with calcium levels <9.0 mg in over 12,000 HD patients, while in another large study from the US involving 40, 538 HD patients, the mortality risk with low serum calcium levels was attenuated after adjusting for confounding variables [18]. In the dialysis outcomes and practice patterns study [32], serum calcium levels < 7.8 mg were associated with lower mortality risk. In agreement with DOPPS, we found a similar trend, though not statistically significant, with serum calcium levels below 2.12 mmol/L.

Hypercalcaemia is an undesirable effect associated with the use of calcium based phosphate binders and vitamin D analogues in controlling secondary hyperparathyroidism. This may likely have accounted for the lower levels of PTH seen in our category of patients with calcium levels above 2.75 mmol/L. Although cinacalcet which is one of the newer drugs that effectively lowers PTH without raising serum calcium levels recently became available in South Africa, it is quite expensive, thus limiting its use to a few of our patients. In addition, the higher mean phosphate level in this group of patients is likely due to the concomitant use of alfacalcidol that enhances intestinal absorption of calcium and phosphate.

A notable finding in the current study is that white patients have a poor survival rate compared to black patients. This finding is consistent with recent emerging data from the USA that reported better survival in black patients compared with white patients on MHD [10]. The reasons underlying this racial survival benefit remain unclear, and several studies have proposed explanations for the better survival of black MHD patients compared to whites. A large US observational study reported that the widely perceived survival advantage for black dialysis patients applies only to older adults, with a reversal of the higher risk of death in the younger age group (<50 years) [33]. This is contrary to several studies including the current study, where the risk persisted after adjusting for the significantly higher mean age in the white patients [34, 35]. Indeed, the better survival in black patients persisted in a study that comprehensively adjusted for demographics and dialysis modality among several other cofounding variables [34].

Another important observation we made in this study was that white patients had significantly higher levels of serum albumin. We expected this to give white patients a survival benefit. However, the reason for this reversal could likely be explained by a finding from a previous study where markers of worse nutritional status (hypoalbuminemia) or smaller muscle mass and increased body fat in African American patients correlated less strongly with mortality than in whites [36]. Additionally, studies have criticized the use of serum albumin in CKD patients as a marker of nutritional status as inappropriate [37, 38]. In fact, the hazard ratio becomes accentuated after adjusting for serum albumin suggesting that the effect of race on mortality is likely to be through other mechanisms besides nutritional status.

In line with previous studies [33, 39], black patients had higher median intact PTH though this was not statistically significant. Some studies have reported survival benefit with active vitamin D therapy and that black patients are more likely to receive active vitamin agents due to higher PTH compared to white patients [40, 41]. However, it is unlikely that treatment with vitamin D alone may explain the racial survival paradox that has existed for several years. Additionally, reports relating to PTH levels have been controversial and studies are divided on which levels are associated with increased mortality. Similar to earlier [17, 18, 42] and more recent studies [10, 43], we did not find significant association of mortality with severe hyperparathyroidism. On the other hand, studies that have shown significant associations are not unified on what levels of PTH are associated with increased mortality. Therefore, randomized control trials are needed to show the effect of treatment on PTH levels that are associated with favorable clinical outcomes.

Our findings should be considered in the context of the following limitations. Firstly, the retrospective nature of this study could not allow us to make causal associations between markers of mineral bone disease and study outcome (death). In addition, the use of a single baseline laboratory measurement precludes the performance of time dependent Cox analysis to account for variations in the biochemical markers on the impact of death over a period of time. However, few studies have shown no significant difference between the baseline and time dependent Cox analysis [12].

Secondly, the relatively small sample size precludes generalizability of our findings to African HD patients. Thus, there is a need for multicentre studies in Africa, to provide robust data on this important clinical entity (CKD-MBD) in African HD patients.

Thirdly, similar to several observational studies we could not account for residual confounding variables. For instance, aside from diabetes mellitus, other comorbid conditions could not be ascertained. However, part of the exclusion criteria was to avoid patients with some coexisting conditions that are known as potential confounders.

The strengths of this study lie in the heterogeneous nature of our study population (black and white patients) in an African setting which has allowed comparisons of data not only for Black Africans with Black Americans, but also between whites in Africa and USA/Europe. To our knowledge, this is the first study in Sub-Saharan Africa that has given important insights regarding the impact of serum alkaline phosphatase, calcium, and race on mortality in African MHD patients.

In summary, high TAP, hypercalcaemia, and white race are associated with increased risk of death in MHD patients, thus, reaffirming the need to pay more attention to the two modifiable risk factors (calcium and TAP) in the management of CKD-MBD.

## Figures and Tables

**Table 1:** Comparisons of baseline characteristics between patients in high TAP and low TAP groups.

Characteristic	All (*N* = 213)	TAP ≤ 112 (*n* = 98)	TAP > 112 (*n* = 115)	*P* value
Age (years)	54.53 ± 15.62	57.3 ± 15.5	51.1 ± 15.1	0.008
Female, *n* (%)	76 (35.7%)	35 (35.7%)	41 (35.7%)	0.25
Diabetes, *n* (%)	56 (26.3%)	27 (27.6%)	29 (25.2%)	0.76
Weight (Kg)	71 ± 9.6	70 ± 9.5	69 ± 9.6	0.53
BMI (Kg/m^2^)	24.7 ± 0.9	24.9 ± 1.0	24.5 ± 1.6	0.83
Dialysis vintage (months)	24 (12–48)	36 (12–60)	36 (12–48)	0.55
Systolic Bp (mmHg)	134 ± 21.8	135.5 ± 19.6	133.5 ± 24.4	0.38
Diastolic Bp (mmHg)	72.0 ± 13.73	70.7 ± 12.0	74.1 ± 13.8	0.86
Haemoglobin (g/dL)	10.3 ± 2.0	10.2 ± 1.9	9.9 ± 2.1	0.10
Potassium (mmol/L)	4.62 ± 0.8	4.6 ± 0.9	4.6 ± 0.8	0.55
Calcium (mmol/L)	2.25 ± 0.14	2.32 ± 0.30	2.34 ± 0.29	0.58
Corrected calcium (mmol/L)	2.40 ± 0.25	2.50 ± 0.22	2.50 ± 0.21	0.42
iPTH (pg/mL)	307 (148–656)	246 (137–527)	325 (152–693)	0.09
Phosphate (mmol/L)	1.59 ± 0.6	1.60 ± 0.6	1.40 ± 0.6	0.07
25-OH vitamin D (ng/mL)	21.16 ± 10.71	20.4 ± 8.8	22.2 ± 12.9	0.83
Alkaline phosphatase (U/L)	112 (74–163)	74 (62–96)	163 (130–223)	<0.001
Albumin (g/L)	31.9 ± 6.0	32.6 ± 5.4	30.3 ± 6.5	0.98
Type of vascular access				
Arteriovenous fistula	129 (60.6%)	65 (66.3%)	64 (55.7%)	0.23
Graft	39 (18.3%)	23 (23.5%)	26 (22.6%)	0.88
Catheter	45 (21.1%)	21 (21.4%)	24 (20.9%)	0.97
Alanine transaminase (U/L)	21.1 ± 8.9	17.6 ± 8.7	22.9 ± 8.8	0.20
Kt/V	1.44 ± 0.28	1.4 ± 0.3	1.4 ± 0.2	0.72
n PCR (g/kg/day)	1.10 ± 0.24	1.02 ± 0.30	1.08 ± 0.27	0.56
T. cholesterol (mmol/L)	4.18 ± 0.91	4.3 ± 0.9	4.1 ± 0.9	0.14
Medications				
Calcium carbonate, *n* (%)	163 (76.5%)	77 (78.6%)	86 (74.7%)	0.74
Alfacalcidol, *n* (%)	137 (64.3%)	61 (62.2%)	76 (66.1%)	0.55
ESA *n* (%)	198 (93.0%)	94 (95.9)	104 (90.4%)	0.50
ESA dose (U/week)	13373 ± 4205	13714 ± 4768	12957 ± 3457	0.53

Continuous variables are presented as means ± standard deviations or median (interquartile range) and categorical data as frequencies (percentages), BP = blood pressure, i PTH = intact parathyroid hormone, TAP = total alkaline phosphatase, ESA = erythropoietin stimulating agent, n PCR = normalized protein catabolic rate, and BMI = body mass index.

**Table 2:** Baseline characteristics of study population by race.

Parameters	All (*n* = 213)	Black (*n* = 120)	White (*n* = 93)	*P* value
Age (years)	54.53 ± 15.62	51.0 ± 14.6	58.7 ± 15.9	<0.001
Haemoglobin (g/dL)	10.3 ± 2.00	9.9 ± 1.98	10.7 ± 1.94	0.004
Systolic Bp (mmHg)	134 ± 21.8	130 ± 20.3	139 ± 22.8	0.98
PTH (pg/mL)	307 (148–656)	327 (137–658)	290 (149–618)	0.97
Calcium (mmol/L)	2.28 ± 0.22	2.26 ± 0.22	2.30 ± 0.21	0.94
Phosphate (mmol/L)	1.59 ± 0.56	1.49 ± 0.57	1.71 ± 0.53	0.004
Albumin (g/L)	31.9 ± 6.0	30.8 ± 6.5	33.04 ± 5.5	0.03
25(OH) vitamin D (ng/mL)	21.16 ± 10.71	20.57 ± 9.79	21.80 ± 11.67	0.77
TAP (U/L)	112 (74–163)	110 (75–151)	115 (71–164)	0.33
T. cholesterol (mmol/L)	4.2 ± 0.8	4.0 ± 0.9	4.1 ± 0.9	0.05
Diabetes, *n* (%)	56 (26.3%)	36 (30.0%)	20 (21.5%)	0.02
Male, *n* (%)	137 (64.3%)	72 (60.0%)	65 (69.9%)	0.07
Kt/V	1.44 ± 0.3	1.41 ± 0.3	1.46 ± 0.30	0.40

Continuous variables are presented as means ± standard deviations or median (interquartile range) and categorical data as frequencies (percentages). BP = blood pressure, TAP = total alkaline phosphatase, and PTH = parathyroid hormone.

**Table 3:** Patient characteristics by serum calcium categories.

Parameters	<2.10 mmol/L (*n* = 31)	2.10–2.37 mmol/L (*n* = 92)	2.38–2.75 mmol/L (*n* = 57)	>2.75 mmol/L (*n* = 33)	*P*-value
Age (years)	50.9 ± 15.0	52.9 ± 15.0	58.3 ± 16.4	56.5 ± 26.1	0.09
Systolic Bp (mmHg)	130.9 ± 18.6	138.8 ± 21.5	139.8 ± 30.7	138.9 ± 21.5	0.18
Diastolic Bp (mmHg)	71.2 ± 15.3	71.7 ± 11.2	76.4 ± 18.9	71.2 ± 11.1	0.38
Haemoglobin (g/dL)	10.8 ± 2.4	10.2 ± 1.9	10.1 ± 1.9	8.15 ± 1.9	0.20
Albumin g/L	32.0 ± 5.2	32.7 ± 6.0	30.5 ± 6.6	29.5 ± 5.0	0.26
T.chol (mmol/L)	4.3 ± 1.0	4.2 ± 0.9	4.2 ± 0.9	4.1 ± 0.9	0.97
25-OH vitamin D (ng/mL)	22.8 ± 9.1	22.0 ± 10.4	18.1 ± 8.1	15.8 ± 3.5	0.11
PTH (pg/mL)	568.8 ± 334.8	458.64 ± 424.4	366.2 ± 405.1	254.0 ± 103.2	0.01
Phosphate (mmol/L)	1.5 ± 0.6	1.6 ± 0.6	1.5 ± 0.5	1.6 ± 0.5	0.66
Creatinine(*μ*mol/L)	822.5 ± 261.0	734.4 ± 283.2	592.5 ± 245.5	489.5 ± 355.7	0.002
Kt/V	1.4 ± 0.2	1.5 ± 0.3	1.4 ± 0.3	1.4 ± 0.4	0.33
Dialysis vintage (months)	31.3 ± 23.0	34.2 ± 23.0	30.9 ± 21.1	30.0 ± 8.9	0.80
Dialysate calcium (mmol/L)	1.65 ± 0.24	1.63 ± 0.14	1.63 ± 0.14	1.54 ± 0.24	0.50
DM, *n*	13	15	17	11	0.40
Medications					
Calcium carbonate *n* (%)	30 (96.8%)	79 (85.7%)	41 (71.9%)	13 (39.4%)	<0.001
Alfacalcidol *n* (%)	28 (90.3%)	63 (68.4%)	35 (61.4%)	11 (33.3%)	<0.001

Continuous variables are presented as means ± standard deviations or median (interquartile range) and categorical data as frequencies (percentages). BP = blood pressure, PTH = parathyroid hormone, *P* values derived by one-way ANOVA, and Kruskal-Wallis tests for continuous variables and Chi-squared for categorical variables. Serum categories based on KDOQI reference range.

**Table 4:** Crude and adjusted hazard ratio (95% CI) of primary outcome by baseline characteristics.

Parameter	Crude HR	95% CI	*P*	Adjusted HR	95% CI	*P*
TAP > 112 U/L	2.20	1.12–4.32	0.02	2.50	1.24–5.01	0.01
Calcium (mmol/L)						
<2.10	0.66	0.32–1.35	0.26	0.97	0.22–4.26	0.97
≥2.10–≤2.37	1.00	Reference				
>2.37–≤2.75	2.31	1.20–4.44	0.02	1.54	0.57–4.18	0.39
>2.75	6.82	1.55–30.1	0.01	6.34	1.40–28.76	0.02
PTH (pg/mL)						
<130	1.00	Reference				
≥130–≤585	1.26	0.57–2.79	0.56	2.77	0.61–12.58	0.19
≥585	1.05	0.44–2.49	0.92	2.22	0.42–11.65	0.35
Phosphate > 1.50 mmol/L	1.09	0.61–1.95	0.77	1.43	0.47-4.40	0.53
25 OH vitamin D ≤ 30 ng/mL	2.21	0.66–7.35	0.19	1.07	0.23–4.79	0.92
White race	1.69	0.95–3.04	0.08	6.88	1.82–25.88	0.004

HR = hazard ratio, CI = confidence interval, TAP = total alkaline phosphate, and PTH intact parathyroid hormone. Adjusted for age, phosphate, calcium, PTH, TAP, diabetes, systolic BP, 25-OH vitamin D, alanine transaminase and albumin, and serum calcium categories based on KDOQI reference range.
